# Health services utilization in the Brazilian Amazon: panel of two cross-sectional studies

**DOI:** 10.11606/s1518-8787.2022056003663

**Published:** 2022-02-09

**Authors:** Gustavo Magno Baldin Tiguman, Marcus Tolentino Silva, Taís Freire Galvão

**Affiliations:** I Universidade Estadual de Campinas Faculdade de Ciências Farmacêuticas Campinas São Paulo Brasil Universidade Estadual de Campinas. Faculdade de Ciências Farmacêuticas. Campinas, São Paulo, Brasil; II Universidade de Sorocaba Programa de Pós-Graduação em Ciências Farmacêuticas Sorocaba São Paulo Brasil Universidade de Sorocaba. Programa de Pós-Graduação em Ciências Farmacêuticas. Sorocaba, São Paulo, Brasil

**Keywords:** Adult, Health Services Accessibility, Health Care Quality, Access, and Evaluation, Health Services Coverage, trends

## Abstract

**OBJECTIVE:**

To investigate the use of health services among adults living in Manaus, Amazonas.

**METHODS:**

This was a panel of two cross-sectional studies conducted in Manaus in 2015 and 2019. Individuals aged ≥ 18 years were selected by probabilistic sampling and interviewed at home. The study outcomes were doctor visits and hospitalizations in the previous 12 months, and unmet surgical needs. Variations between 2015 and 2019 were tested using chi-squared goodness-of-fit test. Poisson regression with robust variance was employed to calculate the prevalence ratios (PR) of the outcomes with 95% confidence intervals (95%CI).

**RESULTS:**

The surveys included 5,800 participants in total. Visits to the doctor decreased from 2015 (78.7%) to 2019 (76.3%; p < 0.001), hospital admissions increased from 2015 (7.9%) to 2019 (11.5%; p < 0.001), and unmet surgical needs decreased in the period (15.9% to 12.1%; p < 0.001). These variations were particularly observed in vulnerable individuals – sicker; poorer; non-whites; and those belonging to lower social classes, with less access to education, formal jobs, and health insurance (p < 0.05). Doctor visits were higher in people with fair health status (PR = 1.09; 95%CI 1.06–1.12), health insurance (PR = 1.13; 95%CI 1.09–1.17), and chronic diseases (p < 0.001) but lower in men (PR = 0.87; 95%CI 0.84–0.90) and informal workers (PR = 0.89; 95%CI 0.84–0.94). Hospitalizations were higher in people with worse health statuses (p < 0.001), without partners (PR = 1.27; 95%CI 1.05–1.53), and with multimorbidity (PR = 1.68; 95%CI 1.33–2.12) but lower in men (PR = 0.55; 95%CI 0.44–0.68), older adults (p < 0.001), informal workers (PR = 0.67; 95%CI 0.51–0.89), and unemployed (PR = 0.72; 95%CI 0.53–0.97). Unmet surgical needs were higher in older adults (p < 0.001), middle-class people (PR = 1.24; 95%CI 1.01–1.55), worse health statuses (p < 0.001), and chronic diseases (p < 0.001) but lower in men (PR = 0.76; 95%CI 0.65–0.86).

**CONCLUSIONS:**

From 2015 to 2019, less people visited the doctor, more were admitted to hospitals, and less were in need of surgery or aware of that need, potentially indicating poorer access to health services.

## INTRODUCTION

The use of health services comprises the direct and indirect contacts with healthcare resources and is associated with individual, financial, cultural, and health system factors^1,2^. Despite some conceptual limitations, access to health care is measured by health services utilization, since the use of such resources demonstrates the access^3,4^.

Universal healthcare is a constitutional right for Brazilian citizens, which was established by the creation of the Brazilian Unified Health System in early 1990’s^5^. Over 70% of the Brazilian population visited a doctor and one-tenth were hospitalized in the previous year up to 2017, with lower utilization in the Northern region^6^. The Brazilian population has relevant gaps in healthcare resources utilization, especially for the most vulnerable individuals^7^. Regional differences also occur: the most developed areas, such as the Southeast and South regions, presented the highest levels of access to health services in a previous national survey conducted in 2013^8^.

The Brazilian Amazon is a heterogeneous setting where large cities coexist with relatively small and isolated villages, in which inequalities in social, economic, and health indicators are present^9^. In 2015, a population-based study in the biggest metropolitan region of the Brazilian Amazon assessed the prevalence of health services usage among adults^10,11^. Self-reported medical consultations were 77% and were higher in women, older people, and those with health insurance, whereas hospitalizations amounted to 7%, were twice as frequent in women compared with men, and thrice as frequent in those who reported very poor health status^10^. Unmet need for surgery affected 14% of the adults and was higher among the elderly, women, and housewives^11^.

Since 2016, Brazil faces political and economic crises, which resulted in the implementation of austerity measures that reduced investments on social and health programs^12^. In 2017, a constitutional amendment established a ceiling for government spending in health, education and social investments for the next 20 years^13^. These austerity measures may significantly reduce primary health coverage, which could cause many avoidable adult and child deaths in the coming years^14^.

A new population-based study conducted in the city of Manaus in 2019 allows a comparison with the results from the previous survey. This analysis could provide important information about the use of health services in the region and the potential effects of Brazilian austerity measures in health care, which can be useful to health policy makers. We aimed to investigate the changes in health services utilization and associated factors among adults from Manaus between 2015 and 2019.

## METHODS

### Study Design

This study was a panel of two cross-sectional studies conducted in 2015 and 2019. The former was carried out in Manaus Metropolitan Region^15^ – which comprises the capital (Manaus) and seven other adjacent municipalities (Careiro da Várzea, Iranduba, Manacapuru, Itacoatiara, Novo Airão, Presidente Figueiredo and Rio Preto da Eva) – and the latter was conducted exclusively in the municipality of Manaus^16^. For the 2015 survey, we only considered the results of Manaus to allow a fair comparison between both studies.

### Setting

The municipality of Manaus is the capital of the state of Amazonas, which is in the North region of Brazil. In 2018, Manaus had 2,145,444 inhabitants, corresponding to more than 50% of Amazonas’ population. The city was in the 8^th^ position for Gross Domestic Product in 2016^17^ and in the 850^th^position on the Human Developing Index in 2010^18^ among Brazilian cities. Manaus concentrates approximately 93% of the physicians from the state of Amazonas, with a density of 2.15 doctors for each 1,000 inhabitants in 2017^19^. Social and economic inequities in the use of health services, and in the consumption and access to medicines characterize the region^20,21^. Historically, the Brazilian Amazon is a region with noteworthy poor health indicators; problems faced by its inhabitants include low income, hazardous work conditions, high violence rates, increased exposure to infectious diseases, lack of household sanitation, and limited access to health services^9^.

### Participants and Sample Size

In both surveys, participants were selected by a three-phase probabilistic sampling stratified by sex and age: census tracts (random), household (systematic), and individual (random)^15,16^. The sample size was estimated in 4,000 participants in the 2015 survey based on 50% of health services usage, confidence level of 95%, absolute precision of 2%, design effect of 1.5, and 2,106,322 adult inhabitants in the metropolitan region^15^. In 2019, the estimated number of participants was of 2,300 based on the 2015 prevalence of healthcare services usage of 20% (10), and considering 2,145,144 adults living in the city of Manaus and similar statistical parameters^16^.

### Variables

The primary outcomes were visits to the doctor and hospitalizations in the previous 12 months and unmet surgical needs (lifetime). Independent variables included: sex (women, men), age group (18–24, 25–34, 34–44, 45–59, and ≥ 60 years old), race/skin color (White, Black, Asian, Brown [Brazilian mixed race], Indigenous), marital status (with partner, without partner), social class (A/B, C, D/E, where A refers to the wealthiest and E to the poorest according to the Brazilian Economic Criteria of each year^22,23^), educational level (higher education or above, high school, elementary school, less than elementary school), occupation (formal job [formal employment relationship which guarantees labor rights and social benefits], informal job [autonomous economic activity without social security or formal relationship with an employer], retired, student/housewife, unemployed), self-perception of health status (good, fair, poor), health insurance (no, yes), and number of chronic diseases (0, 1, ≥ 2).

### Data Sources and Measurement

The primary outcomes were measured by the following questions: “In the last 12 months, how many times have you seen a doctor?”, “In the last 12 months, have you been admitted to the hospital for more than 24 hours?” and “Has any doctor ever said you should have a surgical procedure that you have not done yet?”. The number of doctor visits and hospitalizations was dichotomized to ‘yes’ (≥ 1 visits/hospitalizations) or ‘no’ (0 visits/hospitalizations).

Experienced interviewers were hired and trained by the research authors to proceed with the data collection. Data were obtained from face-to-face interviews with pre-configured questionnaires in the software SurveyToGo (Dooblo Ltd, Israel), using electronic devices (Tab3 SM-T110 Samsung^®^ Galaxy [2015] and Intel TabPhone 710 Pro [2019]). After the interviews, the questionnaires were sent to the research server via internet connection.

### Bias

A pilot study was conducted in both surveys with 150 participants to evaluate the understanding of the questionnaire; these participants were included in the final sample. In each survey, 20% of the interviews were audited by phone. The interviews were recorded and georeferenced by the electronic device.

### Statistical Analysis

Descriptive statistics were used to calculate the absolute and relative frequencies of health services utilization in the previous 12 months. We calculated the absolute and relative variations in the outcomes between 2015 and 2019. The chi-square goodness-of-fit test was used to calculate the significant differences in prevalence between both years. The prevalence ratios (PR) of doctor visits, hospital admissions and unmet surgical needs by each independent variable were calculated using Poisson regression with robust variance with 95% confidence intervals (CI), considering the participants from both surveys. All of the independent variables were included in the adjusted multivariate regression. Wald test was used to assess the significance of the variables in multiple categories. Statistical significance was considered if p-value < 0.05. All analyses were conducted in Stata 14.2 and considered the complex sampling design (svy command).

### Ethics

The Ethics Research Committee from the University of Amazonas approved both studies through the approval letters No. 974.428 from 03 March 2015 and No. 3.102.942 from 28 December 2018. All the participants signed an informed consent form before any study procedure was performed.

## RESULTS

**Figure 1:**
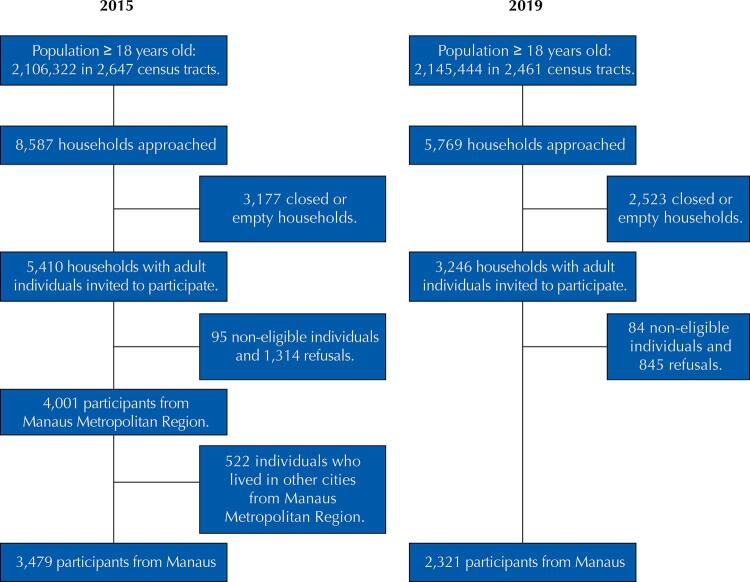
Recruitment processes for the population-based studies in Manaus (2015 and 2019).

In total, 5,800 individuals were included in both surveys (Figure 1). Out of the 3,479 participants interviewed in Manaus in 2015, 78.7% (95%CI 77.4%–80.1%) visited a doctor and 7.9% (95%CI 6.9%–8.8%) were hospitalized in the previous 12 months, while 15.9% (95%CI 14.7%–17.2%) reported unmet surgical need. In 2019, out of the 2,321 participants, 76.3% (95%CI 74.6%–78.1%) consulted a doctor and 11.5% (95%CI 10.1%–12.9%) were hospitalized in the previous 12 months, and 12.1% (95%CI 10.7%–13.5%) failed to have the surgery they needed (Table 1).

**Table 1:** Participants’ characteristics and frequencies of doctor visits and hospital admissions in the previous 12 months and unmet need for surgery in 2015 (n = 3,479) and 2019 (n = 2,321), adjusted for the complex sampling design.

Variables	Participants’ characteristics				Visits to the doctor				Hospital admissions				Unmet need for surgery			
			
	2015		2019		2015		2019		2015		2019		2015		2019	
							
	n	%	n	%	n	%	n	%	n	%	n	%	n	%	n	%
Sex																
Women	1,856	65.1	1,233	64.7	1,538	83.1	992	80.7	186	9.9	175	14.1	330	18.0	165	13.6
Men	1,623	34.9	1,088	35.3	1,139	70.7	742	68.4	67	4.2	75	6.8	188	12.0	99	9.4
Age group (years)																
18–24	716	16.2	405	13.5	525	75.5	284	71.2	62	9.9	50	13.6	56	8.3	24	6.0
25–34	1,010	31.4	586	25.2	752	76.8	440	77.4	67	7.5	82	16.0	114	12.1	50	8.9
35–44	744	22.2	553	25.0	589	80.2	404	74.4	50	7.2	57	10.7	134	19.2	74	14.6
45–59	674	19.0	526	23.9	525	78.6	406	78.3	48	7.6	43	8.7	139	21.0	76	14.7
≥ 60	335	11.2	251	12.4	286	86.4	200	80.0	26	8.1	18	7.2	75	22.6	40	15.4
Race/skin color																
White	545	15.3	283	12.1	406	75.8	220	79.1	40	8.0	31	11.9	76	14.9	27	9.8
Black	241	6.7	215	8.7	199	83.4	142	67.7	20	8.9	16	8.0	32	13.2	18	8.3
Asian	129	3.9	66	2.8	111	86.2	50	77.9	12	10.2	11	16.7	17	14.2	8	14.6
Brown	2,533	73.1	1,677	72.9	1,938	78.5	1,269	77.3	178	7.6	187	11.9	388	16.5	204	13.0
Indigenous	31	1.0	80	3.5	23	77.9	53	66.6	3	11.8	5	6.2	5	19.4	7	8.8
Marital status																
With partner	1,266	37.8	898	39.7	1,020	81.2	683	77.3	89	7.6	76	8.8	220	18.0	107	12.5
Without partner	2,213	62.2	1,423	60.3	1,657	77.3	1,051	75.7	164	8.0	174	13.3	298	14.7	157	11.9
Social class																
A/B	555	14.9	282	11.5	438	80.6	224	80.0	41	8.1	19	7.0	60	11.2	31	11.6
C	2,006	57.5	1,244	53.5	1,524	77.8	929	76.5	136	7.5	136	11.7	307	16.6	132	11.5
D/E	918	27.6	795	35.0	715	79.7	581	74.9	76	8.7	95	12.7	151	17.1	101	13.3
Educational level																
Higher education or above	131	3.9	153	6.9	101	78.8	127	83.9	13	11.7	10	7.1	21	16.6	24	17.3
High school	1,695	47.2	1,171	49.4	1,278	77.3	875	76.3	114	7.4	111	10.7	211	13.3	120	11.2
Elementary school	562	15.6	432	18.0	431	78.4	318	75.9	42	7.9	68	16.1	71	13.5	45	11.4
Less than elementary school	1,091	33.3	565	25.7	867	81.0	414	74.8	84	8.1	61	11.0	215	20.7	75	13.1
Occupation																
Formal job	652	16.6	415	16.1	515	80.2	315	78.4	53	9.1	34	9.2	80	13.3	47	12.3
Informal job	978	26.2	665	27.5	683	72.3	461	70.7	43	4.7	57	9.1	168	18.4	60	9.6
Retired	270	8.7	162	7.6	231	86.4	136	83.7	21	8.3	15	9.5	60	23.2	35	20.5
Student/housewife	1,069	34.2	632	31.3	838	79.9	506	80.9	106	10.4	92	15.2	153	15.4	80	13.3
Unemployed	510	14.3	447	17.5	410	81.5	316	72.2	30	6.1	52	11.8	57	11.7	42	10.2
Health status																
Good	2,243	62.7	1,498	62.5	1,641	75.0	1,059	72.2	130	6.3	114	8.1	226	10.9	107	7.6
Fair	1,012	30.3	671	30.4	852	85.4	548	83.1	92	9.7	106	16.6	236	24.3	113	17.6
Poor	224	7.0	152	7.1	184	82.9	127	84.1	31	14.5	30	19.5	56	25.1	44	28.6
Health insurance																
No	3,027	87.0	1,978	85.3	2,278	77.2	1,449	74.9	215	7.7	215	11.6	461	16.3	226	12.1
Yes	452	13.0	343	14.7	399	89.2	285	84.8	38	9.5	35	11.1	57	13.7	38	12.1
Number of chronic diseases																
0	1,377	37.4	921	37.4	970	72.5	594	66.4	66	5.2	80	9.8	113	9.2	46	5.3
1	989	28.1	682	29.0	775	79.9	507	75.0	64	7.0	70	10.9	130	13.5	66	9.9
≥ 2	1,113	34.5	718	33.6	932	84.6	633	88.6	123	11.4	100	13.9	275	25.3	152	21.7
Total	3,479	100.0	2,321	100.0	2,677	78.7	1,734	76.3	253	7.9	250	11.5	518	15.9	264	12.1

Between 2015 and 2019, doctor visits decreased (−2.4%; p < 0.001), hospital admissions increased (3.6%; p < 0.001), unmet surgical needs decreased (−3.8%; p < 0.001; Table 2). All variables that presented differences (p < 0.05) between both years showed reductions in the prevalence of doctor visits and unmet surgical needs and an increase in hospital admissions (except for those with higher education or above, whose hospitalizations decreased). Medical consultations decreased in this period among women; ages 18–24 years, 35–44 years, and ≥60 years; all races/skin colors except for Whites; formal workers; unemployed; poorer people; those with lower educational achievement; those with better health statuses; and those with none or one chronic disease (p < 0.05). Doctor visits increased only among people with multimorbidity (p = 0.008). Between 2015 and 2019, hospital admissions increased among younger, poorer, Brown, partnerless, less educated, without health insurance, and working informally or unemployed but decreased among people with higher education (p < 0.05). Unmet surgical needs decreased, particularly among those older, poorer, less educated, working informally, with better health statuses, and without insurance (p < 0.05).

**Table 2:** Absolute and relative variations in doctor visits and hospital admissions in the previous 12 months and unmet need for surgery between 2015 (n = 3,479) and 2019 (n = 2,321).

Variables	Visits to the doctor			Hospitalizations			Unmet need for surgery		
		
	Absolute variation (%)	Relative variation	p	Absolute variation (%)	Relative variation	p	Absolute variation (%)	Relative variation	p
Sex									
Women	-2.4	1.0	**0.013**	4.2	1.4	**< 0.001**	-4.4	0.8	**< 0.001**
Men	-2.3	1.0	0.070	2.6	1.6	**< 0.001**	-2.6	0.8	**0.003**
Age group (years)									
18–24	-4.3	0.9	**0.012**	3.7	1.4	0.099	-2.3	0.7	0.083
25–34	0.6	1.0	0.325	8.5	2.1	**< 0.001**	-3.2	0.7	**0.008**
35–44	-5.8	0.9	**< 0.001**	3.5	1.5	**0.005**	-4.6	0.8	**0.001**
45–59	-0.3	1.0	0.429	1.1	1.1	0.619	-6.3	0.7	**< 0.001**
≥ 60	-6.4	0.9	**0.002**	-0.9	0.9	0.590	-7.2	0.7	**0.012**
Race/skin color									
White	3.3	1.0	0.446	3.9	1.5	0.067	-5.1	0.7	**0.011**
Black	-15.7	0.8	**< 0.001**	-0.9	0.9	0.453	-4.9	0.6	**0.036**
Asian	-8.3	0.9	**0.014**	6.5	1.6	0.083	0.4	1.0	0.629
Brown	-1.2	1.0	**0.005**	4.3	1.6	**< 0.001**	-3.5	0.8	**< 0.001**
Indigenous	-11.3	0.9	**0.012**	-5.6	0.5	0.124	-10.6	0.5	**0.016**
Marital status									
With partner	-3.9	1.0	**< 0.001**	1.2	1.2	0.329	-5.5	0.7	**< 0.001**
Without partner	-1.6	1.0	**< 0.001**	5.3	1.7	**< 0.001**	-2.8	0.8	**< 0.001**
Social class									
A/B	-0.6	1.0	0.620	-1.1	0.9	0.402	0.4	1.0	0.912
C	-1.3	1.0	**0.008**	4.2	1.6	**< 0.001**	-5.1	0.7	**< 0.001**
D/E	-4.8	0.9	**< 0.001**	4.0	1.5	**0.001**	-3.8	0.8	**0.001**
Educational level									
Higher education or above	5.1	1.1	0.203	-4.6	0.6	**0.047**	0.7	1.0	0.761
High school	-1.0	1.0	**0.035**	3.3	1.4	**0.007**	-2.1	0.8	**0.002**
Elementary school	-2.5	1.0	**0.016**	8.2	2.0	**< 0.001**	-2.1	0.8	0.061
Less than elementary school	-6.2	0.9	**< 0.001**	2.9	1.4	**0.019**	-7.6	0.6	**< 0.001**
Occupation									
Formal job	-1.8	1.0	**0.028**	0.1	1.0	0.520	-1.0	0.9	0.236
Informal job	-1.6	1.0	0.086	4.4	1.9	**< 0.001**	-8.8	0.5	**< 0.001**
Retired	-2.7	1.0	0.363	1.2	1.1	0.658	-2.7	0.9	0.631
Student/housewife	1.0	1.0	0.918	4.8	1.5	**0.001**	-2.1	0.9	0.056
Unemployed	-9.3	0.9	**< 0.001**	5.7	1.9	**< 0.001**	-1.5	0.9	0.130
Health status									
Good	-2.8	1.0	**< 0.001**	1.8	1.3	**0.037**	-3.3	0.7	**< 0.001**
Fair	-2.3	1.0	**0.006**	6.9	1.7	**< 0.001**	-6.7	0.7	**< 0.001**
Poor	1.2	1.0	0.831	5.0	1.3	0.067	3.5	1.1	0.274
Health insurance									
No	-2.3	1.0	**< 0.001**	3.9	1.5	**< 0.001**	-4.2	0.7	**< 0.001**
Yes	-4.4	1.0	**< 0.001**	1.6	1.2	0.657	-1.6	0.9	0.158
Number of chronic diseases									
0	-6.1	0.9	**< 0.001**	4.6	1.9	**< 0.001**	-3.9	0.6	**< 0.001**
1	-4.9	0.9	**< 0.001**	3.9	1.6	**0.001**	-3.6	0.7	**0.003**
≥ 2	4.0	1.0	**0.008**	2.5	1.2	**0.033**	-3.6	0.9	**0.011**
Total	-2.4	1.0	**< 0.001**	3.6	1.5	**< 0.001**	-3.8	0.8	**< 0.001**

Note: Statistically significant values are shown in bold.

Doctor visits were higher among people with fair health status (PR = 1.09; 95%CI 1.06–1.12), with health insurance (PR = 1.13; 95%CI 1.09–1.17), and with 1 (PR = 1.10; 95%CI 1.06–1.14) or ≥ 2 chronic diseases (PR = 1.17; 95%CI 1.13–1.22). Medical consultations were lower in men (PR = 0.87; 95%CI 0.84–0.90) and informal workers (PR = 0.91; 95%CI 0.87–0.95; Table 3).

**Table 3:** Unadjusted and adjusted prevalence ratios (PR) and 95% confidence intervals (CI) of doctor visits in the previous 12 months in Manaus (n = 5,800).

Variables	PR (95%CI)	p	Adjusted PR (95%CI)	p
Year		0.036		0.063
2015	1.00		1.00	
2019	**0.97 (0.94–0.99)**		0.97 (0.95–1.00)	
Sex		< 0.001		< 0.001
Women	1.00		1.00	
Men	**0.85 (0.82–0.88)**		**0.87 (0.84–0.90)**	
Age group (years)		< 0.001		0.765
18–24	1.00		1.00	
25–34	1.04 (1.00–1.09)		1.03 (0.98–1.08)	
35–44	1.05 (1.00–1.10)		1.02 (0.97–1.07)	
45–59	1.06 (1.01–1.11)		1.00 (0.96–1.06)	
≥ 60	**1.13 (1.08–1.19)**		1.02 (0.96–1.09)	
Race/skin color		0.072		0.371
White	1.00		1.00	
Black	0.99 (0.93–1.06)		1.00 (0.94–1.06)	
Asian	**1.09 (1.01–1.17)**		1.05 (0.97–1.13)	
Brown	1.02 (0.97–1.06)		1.00 (0.96–1.04)	
Indigenous	0.91 (0.80–1.04)		0.91 (0.80–1.03)	
Marital status		0.010		0.114
With partner	1.00		1.00	
Without partner	**0.96 (0.94–0.99)**		0.98 (0.95–1.01)	
Social class		0.128		0.066
A/B	1.00		1.00	
C	0.96 (0.92–1.00)		0.96 (0.92–1.00)	
D/E	0.96 (0.92–1.01)		0.95 (0.91–1.00)	
Educational level		0.139		0.710
Higher education or above	1.00		1.00	
High school	0.94 (0.89–1.00)		1.00 (0.94–1.06)	
Elementary school	0.95 (0.89–1.01)		1.02 (0.95–1.09)	
Less than elementary school	0.97 (0.91–1.03)		0.99 (0.93–1.06)	
Occupation		< 0.001		< 0.001
Formal job	1.00		1.00	
Informal job	**0.90 (0.86–0.94)**		**0.91 (0.87–0.95)**	
Retired	**1.07 (1.02–1.13)**		1.00 (0.94–1.06)	
Student/housewife	1.01 (0.97–1.05)		0.96 (0.92–1.00)	
Unemployed	0.97 (0.93–1.02)		0.97 (0.93–1.02)	
Health status		< 0.001		< 0.001
Good	1.00		1.00	
Fair	**1.14 (1.11–1.18)**		**1.09 (1.06–1.12)**	
Poor	**1.13 (1.07–1.19)**		1.05 (1.00–1.11)	
Health insurance		< 0.001		< 0.001
No	1.00		1.00	
Yes	**1.15 (1.11–1.18)**		**1.13 (1.09–1.17)**	
Number of chronic diseases		< 0.001		< 0.001
0	1.00		1.00	
1	**1.11 (1.07–1.16)**		**1.10 (1.06–1.14)**	
≥ 2	**1.23 (1.19–1.27)**		**1.17 (1.13–1.22)**	

Note: Statistically significant values are shown in bold.

Hospital admissions were higher in 2019 than in 2015 (PR = 1.55; 95%CI 1.30–1.85) for those without partners (PR = 1.27; 95%CI 1.05–1.53), with fair (PR = 1.65; 95%CI 1.36–1.99) and poor health statuses (PR = 2.15; 95%CI 1.63–1.99), and with multimorbidity (PR = 1.68; 95%CI 1.33–2.12), whereas lower in men (PR = 0.55; 95%CI 0.44–0.68), older adults (35–44 years: PR = 0.73; 95%CI 0.56–0.95, 45–59 years: PR = 0.57; 95%CI 0.43–0.77, ≥ 60 years: PR = 0.41; 95%CI 0.27–0.63), informal workers (PR = 0.67; 95%CI 0.51–0.89), and unemployed (PR = 0.72; 95%CI 0.53–0.97; Table 4).

**Table 4:** Unadjusted and adjusted prevalence ratios (PR) and 95% confidence intervals (CI) of hospital admissions in the previous 12 months in Manaus (n = 5,800).

Variables	PR (95%CI)	p	Adjusted PR (95%CI)	p
Year		< 0.001		< 0.001
2015	1.00		1.00	
2019	**1.46 (1.23–1.73)**		**1.55 (1.30–1.85)**	
Sex		< 0.001		< 0.001
Women	1.00		1.00	
Men	**0.45 (0.37–0.55)**		**0.55 (0.44–0.68)**	
Age group (years)		0.041		< 0.001
18–24	1.00		1.00	
25–34	0.93 (0.73–1.18)		0.98 (0.77–1.24)	
35–44	0.77 (0.60–1.00)		**0.73 (0.56–0.95)**	
45–59	**0.72 (0.55–0.94)**		**0.57 (0.43–0.77)**	
≥ 60	**0.69 (0.49–0.97)**		**0.41 (0.27–0.63)**	
Race/skin color		0.642		0.481
White	1.00		1.00	
Black	0.91 (0.61–1.34)		0.86 (0.58–1.27)	
Asian	1.32 (0.84–2.07)		1.18 (0.75–1.84)	
Brown	0.99 (0.78–1.27)		0.88 (0.69–1.12)	
Indigenous	0.84 (0.41–1.73)		0.69 (0.34–1.42)	
Marital status		0.018		0.012
With partner	1.00		1.00	
Without partner	**1.25 (1.04–1.49)**		**1.27 (1.05–1.53)**	
Social class		0.074		0.599
A/B	1.00		1.00	
C	1.17 (0.89–1.54)		1.06 (0.81–1.39)	
D/E	**1.37 (1.02–1.82)**		1.15 (0.85–1.55)	
Educational level		0.114		0.334
Higher education or above	1.00		1.00	
High school	0.95 (0.63–1.43)		1.01 (0.66–1.54)	
Elementary school	1.24 (0.80–1.91)		1.23 (0.78–1.94)	
Less than elementary school	0.98 (0.64–1.50)		1.03 (0.65–1.64)	
Occupation		< 0.001		0.012
Formal job	1.00		1.00	
Informal job	**0.71 (0.53–0.94)**		**0.67 (0.51–0.89)**	
Retired	0.96 (0.66–1.41)		0.97 (0.63–1.51)	
Student/housewife	**1.34 (1.05–1.71)**		0.93 (0.72–1.22)	
Unemployed	0.95 (0.70–1.27)		**0.72 (0.53–0.97)**	
Health status		< 0.001		< 0.001
Good	1.00		1.00	
Fair	**1.78 (1.48–2.13)**		**1.65 (1.36–1.99)**	
Poor	**2.35 (1.80–3.07)**		**2.15 (1.63–2.84)**	
Health insurance		0.399		0.137
No	1.00		1.00	
Yes	1.11 (0.87–1.41)		1.20 (0.94–1.52)	
Number of chronic diseases		< 0.001		< 0.001
0	1.00		1.00	
1	1.22 (0.97–1.54)		1.19 (0.94–1.50)	
≥ 2	**1.77 (1.45–2.18)**		**1.68 (1.33–2.12)**	

Note: Statistically significant values are shown in bold.

Unmet need for surgery was higher in older adults (35–44 years: PR = 1.78; 95%CI 1.38–2.31, 45–59 years: PR = 1.54; 95%CI 1.18–2.01), middle-class people (PR = 1.24; 95%CI 1.01–1.55), those with fair (PR = 1.70; 95%CI 1.45–1.98) and poor (PR = 1.82; 95%CI 1.46–2.28) health statuses, and those with 1 (PR = 1.38; 95%CI 1.12–1.69) or ≥ 2 (PR = 2.16; 95%CI 1.76–2.64) chronic diseases. This outcome was lower in 2019 (PR = 0.75; 95%CI 0.65–0.86) and among men (PR = 0.76; 95%CI 0.65–0.88; Table 5).

**Table 5:** Unadjusted and adjusted prevalence ratios (PR) and 95% confidence intervals (CI) of unmet need for surgery in Manaus (n = 5,800).

Variables	PR (95%CI)	p	Adjusted PR (95%CI)	p
Year		< 0.001		< 0.001
2015	1.00		1.00	
2019	**0.76 (0.66–0.88)**		**0.75 (0.65–0.86)**	
Sex		< 0.001		< 0.001
Women	1.00		1.00	
Men	**0.68 (0.59–0.78)**		**0.76 (0.65–0.88)**	
Age group (years)		< 0.001		< 0.001
18–24	1.00		1.00	
25–34	**1.48 (1.13–1.92)**		1.28 (0.99–1.67)	
35–44	**2.32 (1.80–2.98)**		**1.78 (1.38–2.31)**	
45–59	**2.43 (1.89–3.12)**		**1.54 (1.18–2.01)**	
≥ 60	**2.63 (1.99–3.46)**		1.29 (0.92–1.81)	
Race/skin color		0.150		0.360
White	1.00		1.00	
Black	0.84 (0.60–1.16)		0.86 (0.62–1.19)	
Asian	1.09 (0.72–1.64)		0.93 (0.62–1.41)	
Brown	1.15 (0.94–1.40)		1.08 (0.89–1.32)	
Indigenous	0.91 (0.51–1.61)		0.84 (0.50–1.40)	
Marital status		0.034		0.511
With partner	1.00		1.00	
Without partner	**0.86 (0.76–0.99)**		0.96 (0.84–1.09)	
Social class		0.032		0.144
A/B	1.00		1.00	
C	**1.29 (1.04–1.61)**		**1.24 (1.01–1.55)**	
D/E	**1.36 (1.08–1.71)**		1.20 (0.94–1.52)	
Educational level		< 0.001		0.334
Higher education or above	1.00		1.00	
High school	**0.74 (0.55–0.98)**		0.77 (0.58–1.02)	
Elementary school	0.74 (0.54–1.02)		0.77 (0.56–1.06)	
Less than elementary school	1.07 (0.80–1.43)		0.79 (0.58–1.06)	
Occupation		< 0.001		0.135
Formal job	1.00		1.00	
Informal job	1.15 (0.93–1.41)		0.97 (0.79–1.21)	
Retired	**1.73 (1.35–2.21)**		1.11 (0.81–1.50)	
Student/housewife	1.13 (0.92–1.39)		0.89 (0.71–1.12)	
Unemployed	0.85 (0.66–1.10)		0.77 (0.60–1.00)	
Health status		< 0.001		< 0.001
Good	1.00		1.00	
Fair	**2.26 (1.96–2.60)**		**1.70 (1.45–1.98)**	
Poor	**2.77 (2.26–3.38)**		**1.82 (1.46–2.28)**	
Health insurance		0.150		0.427
No	1.00		1.00	
Yes	0.89 (0.73–1.09)		0.92 (0.74–1.14)	
Number of chronic diseases		< 0.001		< 0.001
0	1.00		1.00	
1	**1.57 (1.28–1.93)**		**1.38 (1.12–1.69)**	
≥ 2	**3.12 (2.62–3.72)**		**2.16 (1.76–2.64)**	

Note: Statistically significant values are shown in bold.

## DISCUSSION

Between 2015 and 2019, doctor visits and unmet surgical needs decreased in Manaus, whereas hospitalizations increased. These variations were particularly pronounced in vulnerable groups, such as poorer and less educated people, all races/skin colors except for Whites, individuals without health insurance, those with informal jobs or unemployed, and people with chronic diseases. Doctor visits were more frequent in people with fair health status, health insurance, and chronic diseases and negatively associated with men and informal workers. Hospital admissions were higher in those without partners, with worse health statuses, and chronic diseases but were lower in men, older adults, informal workers, and unemployed. Unmet need for surgery was higher in older, middle-class, poor health status, and chronically ill individuals and lower in men.

This research was not primarily designed as a comparative analysis but the similarities in the employed methodologies and the outcomes assessments in both surveys enabled the comparison between these two periods for Manaus. Despite the probabilistic sampling method applied in both surveys to minimize selection bias and increase the representativeness of the samples, our sample relied on individuals who were at home at the moment of the interview. All data were based on self-report measures, which are prone to information bias. Despite these limitations, the present analysis is an opportunity to assess the effects of austerity policies on health services utilization in Manaus, implemented after the first survey.

The decrease in doctor visits may be a consequence of the lack of access to primary care and less search for medical assistance for milder diseases, which increased hospital admissions due to worsening of conditions. Our hypothesis is that, as the population becomes sicker with limited access to preventive health care, hospitalization rates rise. In a longitudinal analysis of 5,565 Brazilian municipalities, the economic recession settled in Brazil since 2014 significantly contributed to mortality rate increases, which highlights the importance of health and social protection programs to mitigate health effects, especially in vulnerable individuals^24^. Previous analyses have also found that reducing primary health care coverage with austerity measures and terminating governmental primary care initiatives, such as *Programa Mais Médicos* (More Doctors Program), have potentially increased child and adult mortality in the country^14^. Changes in the Brazilian National Primary Healthcare Policy modified the primary care structure and reduced the Family Health Strategy teams, threatening the interdisciplinarity, accessibility , and community participation of the Brazilian Unified Health System^25^. Social protection, food security, and poverty reduction programs are being dismantled in Brazil, which may also impose health-related hazards to the Brazilian population^26^. Self-medication with antibiotics – potential indicators of poor health status and lack of access to treatments – increased from 2015 to 2019 in Manaus, corroborating this theory^27^.

Unmet need for surgery decreased in this four-year interval. Fewer adults were in need of a previously indicated surgery or aware of this medical need in 2019. Since doctor visits decreased and hospitalizations increased between both years, we hypothesize that Manaus had less diagnoses of health conditions that required surgical interventions and, consequently, less individuals self-reported the unmet surgical demand. Northern Brazil, where Manaus is located, lacks general physicians and surgeons in comparison to other regions since it has the lowest physician density in the country^28^. An ecological and time-series analysis of Brazilian data related to surgical procedures from 2008 to 2016 found that the number of surgeries performed in the North region declined in the period, which contrasts with the rest of the country^29^. This is considerably alarming since untreated surgical conditions burden individuals living in less developed countries, particularly those with the lowest income, those living in rural areas, and those who are marginalized^30^.

Vulnerable populations – such as all races except for Whites, poorer people, less educated people, individuals without health insurance, informal workers or unemployed, and people with chronic diseases – concentrated the poorer outcomes. Important inequities in health services utilization mark Manaus, with long waiting times and considerable discrimination by health professionals, which are significantly higher in socioeconomically disadvantaged people^20,31,32^. The austerity policies adopted in Brazil affect the population unequally, with worse effects to more vulnerable individuals, and hamper the universal, equal, and integral access to health services in Brazil^33,34^. Manaus was one of the Brazilian cities most affected by the COVID-19 pandemic with an explosion in overall mortality at home and on public byways, highlighting the heavy social inequalities and weak effectiveness of governmental policies in the health system^35^.

Individuals with fair health and chronic diseases had more medical appointments, whereas people with fair and poor health and chronically ill had more hospitalizations. A previous nationally representative population-based study conducted in 2013 confirms these findings: negative self-perceptions of health status and chronic diseases were associated with higher seeking for health services and hospitalizations due to worse health conditions^36,37^. Our study also found that unmet surgical needs were more frequent in these individuals. A cross-sectional study with 11,378 Korean adults in 2016 found a higher proportion of unmet medical needs, including surgery, among those with poor health status and with chronic diseases^38^. This finding suggests inequities in the access to these procedures among sicker individuals, who are in higher need of assistance.

Having a health insurance was associated with higher rates of doctor visits. The Brazilian population may face barriers and negative experiences while accessing the public health system, which might constitute important reasons for the pursuit of private health care alternatives^39^. A limited proportion of Manaus Metropolitan Region’s inhabitants has access to private health insurance since this prevalence was 13% in the 2015 survey and was lower among poorer people and those with less schooling^40^.

Medical consultations, hospitalizations and unmet demand for surgery were lower among men in comparison to women. A population-based study conducted in the South of Brazil with 1,297 individuals in 2016 observed that men were less likely to have consulted a physician in the last 12 months when compared to women^41^. A plausible explanation for these findings is that men tend to seek for health services and to care for their health less than women, mainly due to social and cultural influences^42^. Unhealthy diet and lifestyles such as tobacco and alcohol use, and underutilization of health services, disproportionally affect men as a consequence of gender differences dictated by society and the predominant norms of masculinity in health-seeking behaviors^45^. In contrast, women tend to be more health-conscious and engaged in preventive behaviors than men^46^.

Hospitalizations were lower among older adults, while unmet need for surgery was higher in this group. A previous analysis of Brazilian surveys showed a positive trend in self-perception of health as good or excellent among the aging people between 1998 and 2008^47^. This gain in health status and quality of life among the elderly may lead to more self-care and higher seeking for preventive care, resulting in less hospitalizations^48^. Among the Brazilian elderly, the demand for surgical care increased between 1998 and 2013 – a period that also experienced reductions in the availability of surgical beds^49^.

Doctor visits and hospitalizations were lower among informal workers and unemployed people. Our findings reinforce the results from the Brazilian National Household Sample Survey from 2008, which found that informal workers and unemployed individuals showed worse health statuses, greater difficulty in accessing health services, and lower health services seeking compared with formal workers^50^. Previous data from Manaus Metropolitan Region also suggest that the health-related quality of life is lower among informal workers when compared to those with formal jobs^51^.

## CONCLUSIONS

Between 2015 and 2019, visits to the doctor decreased whereas hospital admissions increased in Manaus, also less people were in need of surgery or aware of this need, which potentially indicates poorer access to health care and worsening of diseases. Socioeconomically disadvantaged and sicker individuals were those mainly affected by these outcomes, which may represent early effects of austerity policies in course in Brazil.
